# Investigation of Neuromuscular Activation in Older Female Adults during a Dynamic and Challenging Virtual Reality Activity: A Cross-Sectional Study

**DOI:** 10.3390/jfmk9030143

**Published:** 2024-08-24

**Authors:** Konstantina Intziegianni, Marc Sarens, Olia Tsivitanidou, Louis Nisiotis, Katalin Kovacs, Eniko Nagy, Efstathios Christodoulides

**Affiliations:** 1 School of Science, University of Central Lancashire Cyprus (UCLan Cyprus), 7080 Pyla, Cyprus; otsivitanidou@uclan.ac.uk (O.T.); lnisiotis@uclan.ac.uk (L.N.); echristodoulides@uclan.ac.uk (E.C.); 2 University College of Physical Education and Sports Recreation, Erasmus Hogeschool Brussel (EHB), 1070 Anderlecht, Belgium; 3 INQUIRIUM Ltd., 2333 Nicosia, Cyprus; 4 Faculty of Education and Psychology, Institute of Health Promotion and Sport Science, Eotvos Lorand University, 1053 Budapest, Hungary; kovacs.katalin@ppk.elte.hu (K.K.); eniko.nagy@trebag.hu (E.N.)

**Keywords:** electromyography, EMG amplitude, aging, neuromuscular activation, physical activity

## Abstract

The use of virtual reality (VR) in older adults promotes improvements in mobility, strength, and balance. Changes in neuromuscular activation have been found to be associated with these improvements; however, during VR activities, this aspect has not been thoroughly investigated. The aim of this study was to investigate neuromuscular activation among older female adults during VR activities. Sixteen older female adults, with the use of VR, performed dynamic punching movements involving elbow flexion/extension for one minute, and the muscle activation of the bicep brachii was recorded with electromyography (EMG) and normalized to the maximal voluntary isometric contraction of elbow flexion. The one-minute activity was divided into three time phases: 0–10 s, 25–35 s, and 50–60 s. The five highest EMG amplitude values (%) in each phase were selected and averaged. Differences between phases were analyzed using repeated ANOVA (α_adj_ = 0.017). The EMG amplitude for the first phase was 39.1 ± 2.6%, that for the second phase was 44.8 ± 3.0%, and that for the third phase was 49.6 ± 3.1%. Statistically significant differences were found in all phases, with the first phase demonstrating a lower EMG amplitude (%) compared to the second (*p* = 0.002) and third phases (*p* = 0.000). The third phase demonstrated a higher EMG amplitude (%) compared to the second phase (*p* = 0.025). Engagement in VR activities can have significant effects on neuromuscular activation in older female adults, with our findings revealing a significant increase in the EMG amplitude within one minute of commencing a dynamic and challenging activity such as virtual boxing.

## 1. Introduction

Being physically active can be considered as a protective mechanism against many chronic diseases [1] such as cardiovascular and metabolic diseases, as well as musculoskeletal and mental disorders [2]. Despite the many health benefits of being physically active, the levels of inactivity among older individuals are very high, with the prevalence being the highest among adults who are 65 years old and older [3]. Moreover, inactivity seems to be gender-specific, as older females tend to participate less than older males in regular sustained activity [4]. Previous studies investigating gender discrepancies in physical activity have found that older females engage in physical activity with less frequency, for shorter durations, and with lighter intensity than males [4,5,6].

The combination of aging and inactivity increases both the risk of mortality and prevalence of many chronic diseases and negatively influences the quality of living [7]. According to Milanovic et al., aging results in an increase in body fat, a reduction in muscle strength, and lower levels of flexibility, agility, and endurance [8]. In addition, inactivity in older adults leads to a 40% reduction in muscle mass and a 10–40% reduction in joint motion, a 30% decrease in muscle mass, and a significant reduction of 30% in aerobic capacity [9]. Muscle mass and strength decline by 30–50% between the ages of 30 and 80 years old [10], with the rate of decline per decade being 12–14% after the age of 50 [11]. The decline of these physiological functions is associated with slower walking speeds and difficulties rising from a seated position and balancing [12].

Changing lifestyle behavior in older adults in order for them to become more physically active is not an easy task, and numerous factors contribute to this issue [13]. According to Chao et al., involvement in moderate physical activity is considered by many older adults as a waste of time [14], and proximity to exercise facilities was found to significantly influence the level of activity [15,16]. Other barriers include safety and fear of injury, as older adults often believe that they are too old and fragile for physical activity [17]. Loss of interest is another common factor causing older individuals to terminate their regular exercise program, as conventional exercises are considered monotonous and boring [18].

Recent advancements in technology such as the use of virtual reality (VR) [19,20] confronts many barriers set by older adults with regard to participation in physical activity [13,17]. VR allows individuals to be involved in physical activities within a challenging, enjoyable, and safe environment within the comfort of their own homes [19,21,22]. Studies have suggested that the usage of VR by older adults can promote improvements in mobility [23,24], muscular strength [25], balance control [26] and reaction time [24]. According to the findings of recent studies, the usage of VR by older stroke patients is associated with improved balance and gait ability, highlighting its potential application for rehabilitation purposes [20,27]. However, despite the many benefits for physiological function that the use of VR has, its acute effects on the neuromuscular activation responsible for these changes during VR activities have not yet been investigated.

Surface electromyography (EMG) is a method used for recording the electrical signals emanating from the muscles and quantifying the level of muscular activation during contraction. EMG amplitude (%) is a parameter often used to estimate the level of muscle activation during an activity in relation to its maximum isometric effort [28,29,30]. EMG amplitude is a property that is influenced by several factors such as the firing rates and recruitment characteristics of motor units (MUs) and is strongly related to muscle force [31]. The interaction between the recruitment and firing rate of MUs is muscle- and task-dependent [32], with smaller and slower MUs recruited at lower force levels and larger and faster MUs recruited as force demand increases, thus increasing the EMG amplitude [33]. During sustained exercise, performance fatigability [34] is known to influence the EMG signal, with an increase in its amplitude and a decrease in its characteristic spectral frequencies [35]. In light of the aforementioned benefits and challenges associated with physical activity in older adults and the promising role of VR in enhancing exercise engagement and effectiveness, the aim of the present study was to investigate neuromuscular activation in older female adults during a dynamic and challenging virtual reality activity. The hypothesis of this study is that the EMG amplitude will increase as the length of the activity increases.

## 2. Materials and Methods

### 2.1. Participants

Sixteen older female participants with no previous experience in VR technology volunteered to participate the present study (age: 72 ± 5 y; height: 165 ± 10 cm; weight: 68 ± 8 kg). Participants were randomly recruited through advertisements on social media and posters on outdoor trails and in fitness parks. Additionally, contact lists of participants from previous unrelated projects were utilized, along with snowball sampling, to further recruit participants for this study. The inclusion criteria for the participants required them to be healthy based on their medical history, have normal or corrected to normal eyesight, be pain-free, and be within an age range of 65–80 years. Participants who did not meet the inclusion criteria were excluded from this study. Prior to the recruitment process, ethical approval was obtained from the Research Ethics Committee of the Faculty of Pedagogy and Psychology (ELTE) of Eotvos Lorand University, which granted permission for the study to be performed with the reference number 2023/381. The study was conducted according to the international standards for the use of human subjects, as described within the Declaration of Helsinki [36]. All participants completed an informed consent form. A post hoc analysis was performed in G*power software (version, 3.1.9.7) to compute the achieved power based on the given sample size of the group, the number of measurements (3), and the calculated effect size from partial η^2^ of 0.599, resulting in an effect size f(U) of 1.22 and an achieved power (1−β err prob) of 0.99.

### 2.2. Study Design

A cross-sectional experimental design with a within-subject comparison [37] was used in the present study. Participants (*n* = 16) were asked to visit the laboratory once to perform a boxing activity for one minute within a VR environment while, simultaneously, their muscle electrical activity was recorded, divided into three phases, and compared. To evaluate the reproducibility of the participants’ measurements, some participants (*n* = 11) agreed to visit the laboratory twice (test, re-test design). As reproducibility was not the focus of this study, the reliability results are reported in the Materials and Methods section to support the methodology and approach used.

### 2.3. Instruments and Protocol

A standardized warm-up of 10 repetitions of elbow flexion/extension for both arms was performed with 2 kg dumbbells. Participants’ skin over the muscle belly of the bicep brachii on their dominant side was cleaned with alcohol and shaved if excess hair was present. An electromyography sensor (Delsys Inc., Boston, MA, USA, Trigno Avanti Sensor) was placed in parallel to the direction of the muscle fiber arrangement according to the SENIAM guidelines [38]. To ensure consistency and accurate sensor placement, the operator was always the same. Participants were first instructed to perform maximal voluntary isometric contraction (MVIC) of elbow flexion in a seated position with their hip flexed at 90°. The elbow joint was placed at 45° of flexion on a fixed surface with the use of an electrical goniometer and their arm and forearm in a supinated position. A fixed non-moving handle was placed on each participant’s wrist. After one test trial, participants were instructed to perform 5 s of maximal isometric contraction of elbow flexion while, simultaneously, the electrical muscle activation of the bicep brachii was recorded via EMGworks Acquisition software (Delsys, v4.8.0). The instructions given to the participants were to pull the non-moving handle as hard as possible towards them without changing their elbow joint position or lifting their elbow from the surface. Verbal encouragement was given to all participants.

**Figure 1:**
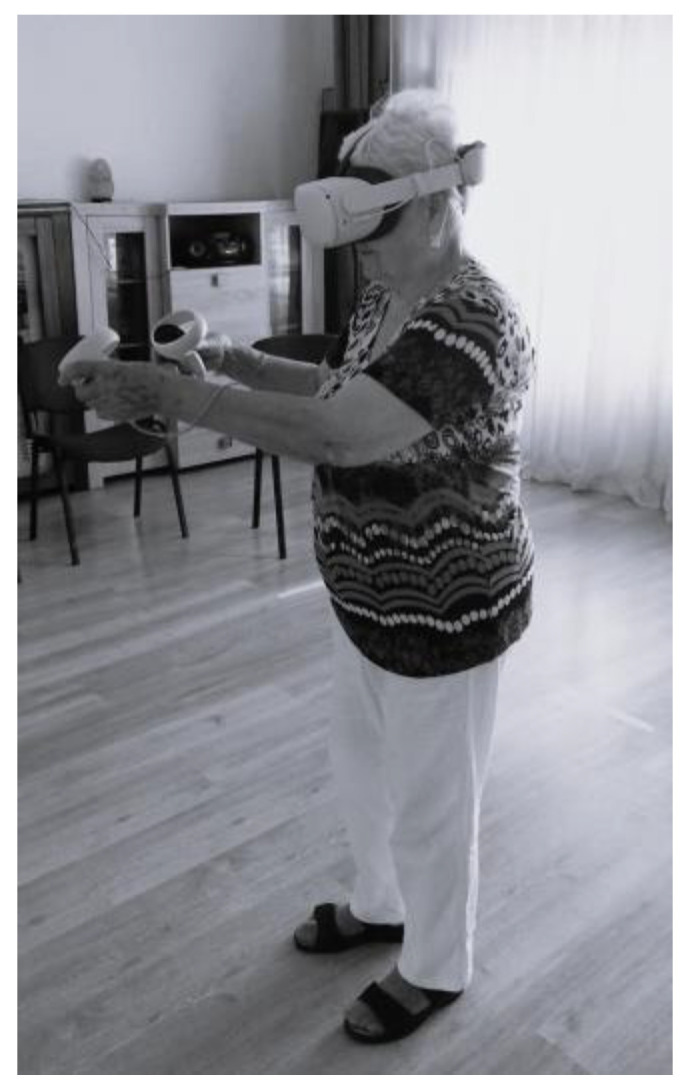
Participant engaging in the virtual reality environment.

After 2 min of rest, participants were then introduced to the VR technology (Meta Quest 2). The Meta Quest 2 headset was carefully placed upon participants’ heads and the controllers were placed into their hands (Figure 1). The Meta Quest 2 (consumer edition, Meta, Menlo Park, CA, USA) is a head-mounted display (HMD) that is able to immerse the user in a virtual environment. It can detect changes in the orientation of the head as well as the hands using three sensors: a gyroscope, an accelerometer, and a magnetometer [39]. This HMD is a “standalone” version, allowing it to operate without an external computer. This means that the device is untethered, therefore giving the user more freedom to move.

After gaining familiarity with the environment of virtual reality, a game called “Knockout League” was introduced to the participants. The specific game/application was chosen specifically as it provided the participants with a challenging, simple, safe, and fun environment to exercise, with the aim being to punch the boxing bag within the VR environment. Participants were instructed to punch the boxing bag as hard as possible within the virtual environment for one minute, while the electrical activity of their bicep brachii muscle was recorded simultaneously. During the use of virtual reality, the investigators ensured that the space was clear of any obstacles and were always at a safe distance observing the participants for their safety.

### 2.4. Outcome Variables and Data Analysis

To quantify the level of muscle activation during the VR activity, the data were normalized with the data collected during the maximum isometric phase [40]. The EMG data were collected during the measurement using the Delsys EMG Trigno Wireless system (Delsys Inc, Boston, MA, USA) which was connected to a PC running Delsys EMGworks Acquisition software (version 4.8.0) and processed for further analysis with the software Delsys EMGworks Analysis (version 4.7.3.0) with an analog band-pass Butterworth fourth-order filter at 20–450 Hz [41] and a sampling frequency of 1259.26 Hz. The root mean square (RMS, (mV)) computations were performed and normalized against the peak RMS (mV) value of the MVIC trial across the entire 5 s duration [40] using an RMS window length of 0.125 s with 0.0625 s of overlap. The main outcome variable was the EMG amplitude [%] as it provides a measure of the muscle activation magnitude of an undergoing activity in relation to its maximum muscle activation [28,29,30].

**Figure 2:**
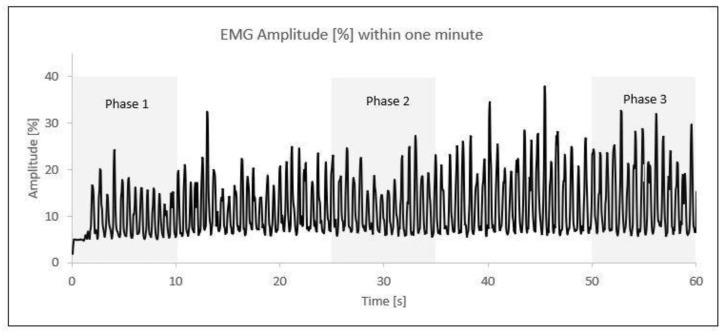
EMG amplitude (%) phases within one minute of elbow flexion/extension; each peak represents one contraction. An example from one participant.

After the normalization of the data, the one-minute activity was divided into three time phases. The first phase comprised the initial 10 s (0–10 s), the second phase included the middle 10 s (25–35 s), and the third phase covered the final 10 s (50–60 s). Within each 10 s phase, the five highest amplitude values (%) were selected and averaged into a single value (Figure 2).

### 2.5. Reliability of the Approach Used

**Figure 3:**
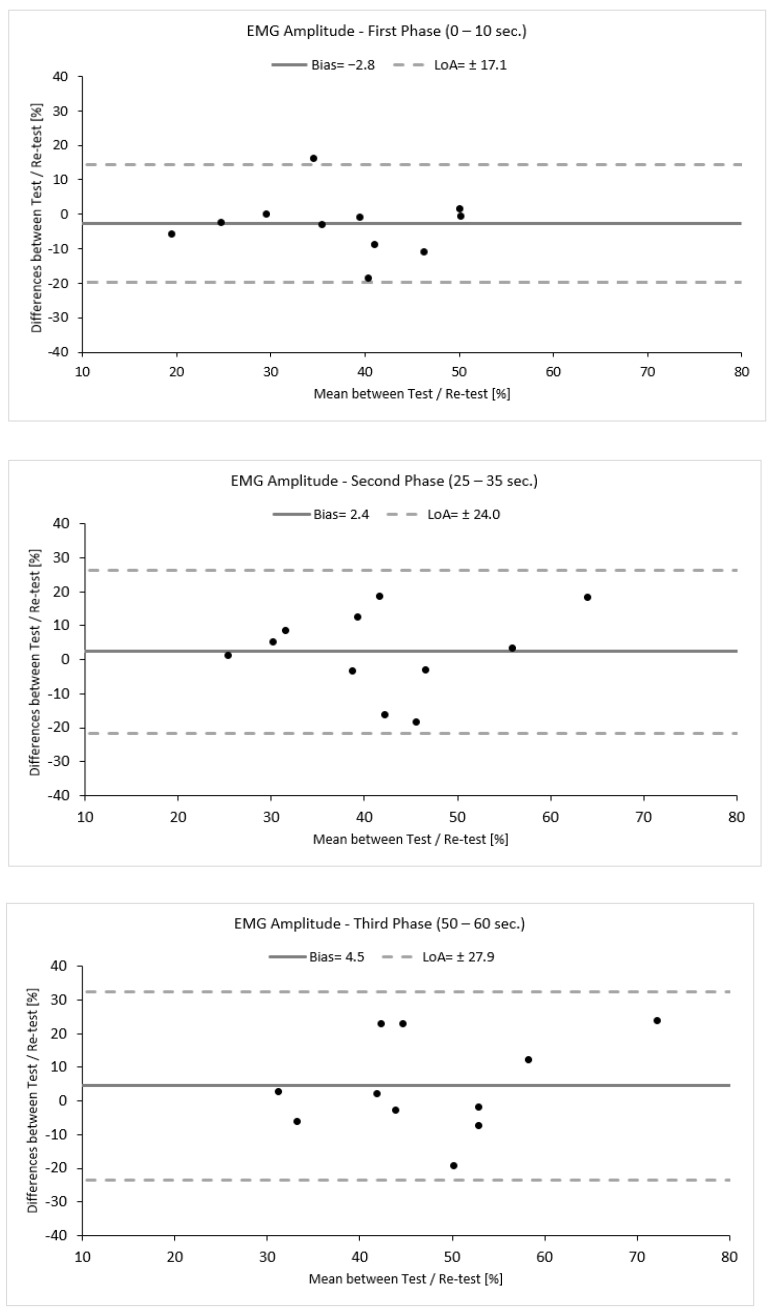
Bland–Altman plots demonstrating the agreement between the test and re–test for each phase. Black dots represents individual data points.

To evaluate the reproducibility of this approach, a small-scale reliability study was performed within this study. Eleven out of sixteen participants revisited the lab within one week. Reliability was assessed by means of intra-class correlation coefficient analysis (ICC, 2,1) with a 95% confidence interval (CI: 95%). An ICC value of ≤0.50 was considered low, and ICC value of 0.50 to 0.75 was considered moderate, an ICC value of ≥0.75 was considered good, and an ICC value of ≥0.90 was considered excellent [42]. The agreement between the measurements was verified qualitatively using Bland–Altman analysis (bias ± limits of agreement (LoA)) and was calculated using the following equation: Bias ± 1.96 × SD (Figure 3) [43].

Variability was calculated as the absolute difference between the two measurements for each phase divided by their average and expressed as a percentage (%). Additionally, to provide an estimate of the precision of measurement, the standard error of measurement (SEM) was calculated using the following equation: SEM = SD × $\sqrt{1-ICC}$ [43]. SEM was further used to calculate the minimal detectable change (MDC), which is the minimal amount of change that a measurement must show to be greater that the within-subject variability and measurement error, calculated according to the following equation: MDC = 1.96 × SEM × $\sqrt{2}$ [44]. The reliability values are presented in Table 1 and Figure 3. The data analysis demonstrated a good to moderate reliability with increasing levels of variability (ICC 2,1, TRV %) accompanied by a high standard deviation, indicating high variability between participants. Moreover, the first phase seemed to be more reproducible and precise and have fewer errors compared to the second and third phases, as indicated by the statistical tests used and by the increasing values of SEM (%) and MDC (%) (Table 1, Figure 3).

### 2.6. Statistics

After confirming the normal distribution of the data via the Shapiro–Wilk test (*p* > 0.05), the data were analyzed descriptively (mean ± standard deviation). To evaluate differences between the three phases in EMG amplitude (%), repeated measures ANOVA was used followed by Bonferroni’s post hoc correction test with an adjusted α level based on the 3 comparisons made (0.05/3 = 0.017). Thus, the statistically significance level was set to α_adj_ = 0.017. Effect sizes were calculated using Cohen’s d [45] for each post hoc test using *t*-test pairwise comparisons. Based on Cohen’s d, an effect size between 0.2 and 0.49 indicates a small effect, an effect size between 0.5 and 0.79 indicates a moderate effect, and an effect size above 0.80 indicates a large effect. The effect size of the ANOVA repeated test was calculated based on the formula provided by Cohen, where the Eta^2^ is converted into Cohen’s f as follows: *f* = $\sqrt{\left(\eta^{2}/(1-\eta^{2})\right)}$ [45]. Based on Cohen’s f, an effect size of *f* = 10 indicates a small effect, *f* = 25 indicates a medium effect, and above *f* = 40 indicates a large effect size. All statistical analyses were performed using SPSS (SPSS Statistics 26, IBM, Armonk, NY, USA) and Microsoft Excel (2016 MSO, Version 2402).

## 3. Results

**Figure 4:**
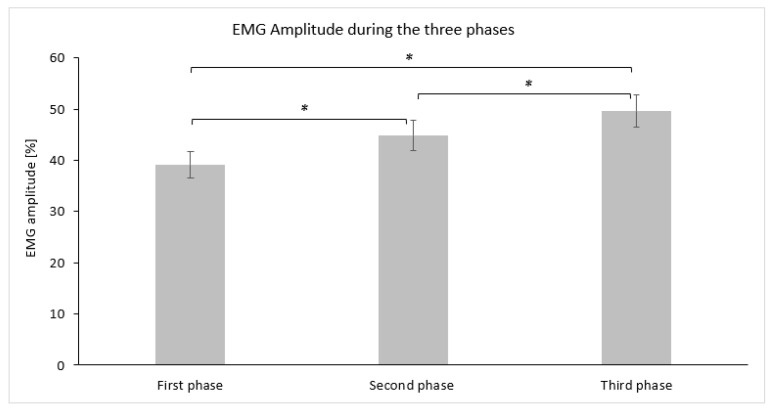
Bar graph demonstrating the mean and standard deviation of the EMG amplitude (%) for each phase. Asterisks (*) indicate statistically significant differences (α_adj_ = 0.017).

The average descriptive values (mean ± SD) for EMG amplitude (%) measured for each phase are graphically demonstrated in Figure 4. Statistically significant differences in EMG amplitude (%) were observed using repeated measures ANOVA (F(2,30) = 22.431, *p* = 0.000, η^2^ = 0.599, Cohen’s *f* = 1.22). Post hoc comparisons using Bonferroni adjustment revealed that the first phase demonstrated a lower EMG amplitude (%) at a statistically significant level (*p* ≤ 0.017) compared to the second phase, while the third phase also displayed a large effect size (Table 2). The third phase demonstrated a higher EMG amplitude (%) compared to the second phase at a statistically significant level (*p* ≤ 0.017) with a moderate effect size (Table 2).

## 4. Discussion

The present study aimed to investigate neuromuscular activation in older female adults by evaluating the EMG amplitude in response to a dynamic VR environment simulating boxing. The findings indicate that one minute of engagement in a dynamic virtual environment simulating boxing can produce significant effects on the activation of the muscle involved, as EMG amplitude significantly increased throughout the three phases. Thus, these findings confirm the initial hypothesis according to which the EMG amplitude (%) will increased as the length of the activity increases. The EMG amplitude, however, is a parameter which can be influenced by many factors [31], and interpretation should be carried out with caution, taking into consideration possible interferences such as the protocol and normalization methods used [46].

The reproducibility of an EMG protocol depends mainly on the task and muscle assessed [47], with the literature presenting mixed results ranging from poor to excellent reliability [48]. Buckthorpe et al. found the absolute EMG amplitude to be highly variable for individuals between measurement sessions for both maximal and explosive voluntary contractions; however, after normalization, the in-between variability was reduced [49]. On the other hand, Lanza et al. found excellent reliability and agreement between days for the EMG parameters, highlighting that care is required when comparing between studies that used different EMG amplitude normalization procedures [46]. The most commonly used and recommended method for normalization is measuring the (peak) activation levels during maximum isometric contraction [40,46], a method that was also applied in this study. In the present study, the one-minute activity was divided into three phases, with reliability being phase-dependent. The first 10 s period was the most reproducible, showing lower levels of variability compared to the middle and last 10 s. This increase of ~5% in variation in the two last phases could be explained by several factors, such as the increase in motor units’ recruitment and the possible influence of fatigue. Previous studies have shown that these aspects can lead to variations in EMG amplitude; however, with proper normalization techniques, variation levels can be kept low [40,46].

EMG amplitude values are expressed as a percentage of their value in relation to the maximal voluntary isometric contraction of the muscle of interest, which is set to 100% [50]. In the present study, there was a statistically significant difference between the three phases, with the first 10 s recording the lowest EMG amplitude of 39% and the last 10 s recording the highest EMG amplitude of 50%. This finding indicates an increase of more than 10% in EMG amplitude and the ability to reach 50% of their maximum level of activation within one minute of engaging in VR activities. This finding has important implications regarding the usage of VR, as previous studies have found that activities influencing neuromuscular activation contribute to an increase in muscle strength and can improve balance and coordination in older adults [51,52,53,54].

Usually, the time spent performing VR activities varies from 10 min to an hour depending on the intensity and purpose [19]. In the present study, a one-minute activity was chosen to minimize the effects of fatigue and possible compensating mechanisms and maximize the effect that a challenging and dynamic VR activity will have on the neuromuscular activation of older female adults. Previous studies indicated that the use of VR in older individuals has many positive effects in improving balance [26], strength [25], mobility [23,24], and gait [27], and even decreasing the risk of falls [55]. VR interventions in older adults were also found to produce even better outcomes compared to conventional exercises [20]. However, the underlying possible mechanism responsible for these improvements in physiological parameters was not investigated. To our knowledge, this is one of the first studies to investigate neuromuscular activation during the use of VR in healthy older adults.

In the present study, only older female adults participated, and thus the results cannot be generalized to the older male population. The use of EMG as the only indicator of performance should be used with caution, as it represents only the electrical function of the muscle and not the mechanical function [56]. An integrated analysis of kinematics and strength measurements could have provided a more accurate representation of the participants’ performance [56]. Even though participants were instructed on how to perform the punching movement, variations during the execution in terms of technique and speed of the movement between participants might have affected the results. Lastly, the rate of perceived excursion would have provided insights into the participants’ perceptions regarding their performance, an aspect which was not evaluated.

## 5. Conclusions

The present study revealed a significant increase in the EMG amplitude during one minute of a dynamic and challenging activity, namely VR boxing, indicating that engagement in VR activities can have a significant influence on neuromuscular activation in older female adults. These findings support and promote the use of VR in elderly populations as a tool for promoting physical activity, health, and wellbeing. However, one must consider that neuromuscular activation as the only indicator of performance should be used with caution and that a combination with kinematic analysis and strength measurements would provide a more accurate representation of the participants’ performance. Furthermore, future research should focus on determining the long-term effects on neuromuscular activation that older adults may experience when training in VR environments, as well as the effects on performance parameters such as strength, balance, and mobility.

## Figures and Tables

**Table 1:** EMG amplitude (%) reproducibility between the test and re-test.

Phases	ICC [2,1]	TRV [%]	SEM [%]	MDC [%]
First phase	0.80	17 ± 17	4.8	13.2
Second phase	0.71	23 ± 15	6.7	18.6
Third phase	0.62	23 ± 18	8.3	23.0

First phase: 0–10 s; second phase: 25–35 s; third phase: 50–60 s. Measures of reliability: ICC = Intraclass correlation coefficient; TRV = test–retest variability; SEM = standard error of measurement; MDC = minimal detectable change.

**Table 2:** ANOVA repeated results and Cohen’s d effect size calculations.

Pairwise Comparisons	*p* Value	Cohen’s *d*
P1 vs. P2	0.002 *	1.1
P1 vs. P3	0.000 *	1.5
P2 vs. P3	0.025 *	0.7

First phase (P1): 0–10 s; second phase (P2): 25–35 s; third phase (P3): 50–60 s. * Statistically significant differences (α_adj_ = 0.017) determined by repeated ANOVA followed by Bonferroni’s post hoc correction test. Cohen’s *d* effect size: 0.2–0.49 = small effect; 0.5–0.79 = moderate effect; above 0.80 = large effect.

## Data Availability

The raw data supporting the conclusions of this article will be made available by the authors upon request.
